# Energy balance, metabolism and cancer prevention: mechanistic insights from transdisciplinary studies

**DOI:** 10.1186/1753-6561-6-S3-O11

**Published:** 2012-06-01

**Authors:** Stephen D Hursting

**Affiliations:** 1 Department of Nutritional Sciences, The University of Texas at Austin and Department of Molecular Carcinogenesis, the University of Texas MD Anderson Cancer Center, USA

## Introduction

The prevalence of obesity, an established risk factor for many cancers, has risen steadily for the past several decades in the US and many other countries. Unfortunately, the mechanisms underlying the obesity and cancer connection are not well understood, and new targets and strategies for offsetting the impact of obesity on cancer risk and/or progression are urgently needed.

## Methods and results

We have established that calorie restriction (CR), the most commonly recommended dietary strategy for preventing or reversing obesity, inhibits spontaneous, transplanted and chemically induced tumors in a variety of animal models. In contrast, diet-induced obesity enhances tumorigenesis in many of these same models. We have also shown in a series of transgenic model systems and microarray studies that the insulin/insulin-like growth factor (IGF)-1 pathway appears central to many of the anti-cancer effects of CR and pro-cancer effects of obesity. Using AZIP/F1 transgenic mice (which lack white adipose tissue but have high levels of insulin, IGF-1 and inflammatory markers), and liver-specific IGF-1-deficient mice, we have reported that elevated IGF-1/insulin resistance/inflammation (which typically accompany obesity), independent of the adipose tissue per se, appear to be the important targets for disrupting the obesity-cancer link. Also, genetic and pharmacologic (rapamaycin) approaches suggest the Akt/mammalian target of rapamycin (mTOR) pathway (downstream of insulin and IGF-1 receptors) provides an important target for disrupting the obesity-cancer link.

## Conclusion

A better understanding of the mechanisms underlying the energy balance-cancer link will facilitate the development of novel prevention and treatment strategies for offsetting the effects of obesity on cancer.

## Funding

NCI R01CA135386 and the Breast Cancer Research Foundation.

